# Development of an Electrochemical Immunosensor for Specific Detection of Visceral Leishmaniasis Using Gold-Modified Screen-Printed Carbon Electrodes

**DOI:** 10.3390/bios10080081

**Published:** 2020-07-23

**Authors:** Beatriz R. Martins, Yanne O. Barbosa, Cristhianne M. R. Andrade, Loren Q. Pereira, Guilherme F. Simão, Carlo J. de Oliveira, Dalmo Correia, Robson T. S. Oliveira, Marcos V. da Silva, Anielle C. A. Silva, Noelio O. Dantas, Virmondes Rodrigues, Rodrigo A. A. Muñoz, Renata P. Alves-Balvedi

**Affiliations:** 1 Institute of Biological and Natural Sciences, Federal University of Triângulo Mineiro, Uberaba-MG 38025-180, Brazil; biaroma_95@hotmail.com (B.R.M.); yanne.way@hotmail.com (Y.O.B.); carlo.oliveira@uftm.edu.br (C.J.d.O.); robson.junior@uftm.edu.br (R.T.S.O.J.); virmondes.rodrigues@uftm.edu.br (V.R.J.); 2 Institute of Health Sciences, Federal University of Triângulo Mineiro, Uberaba-MG 38025-180, Brazil; cristhianne_m@hotmail.com (C.M.R.A.); lorenbiomedica@gmail.com (L.Q.P.); dalmo@mednet.com.br (D.C.); marcosuftm@gmail.com (M.V.d.S.); 3 Institute of Technological and Exact Sciences, Federal University of Triângulo Mineiro, Uberaba-MG 38025-180, Brazil; guilhermefelipesimao@gmail.com; 4 Institute of Physics, Federal University of Alagoas, Maceio-AL 57072-970, Brazil; acalmeida@fis.ufal.br (A.C.A.S.); noelio@fis.ufal.br (N.O.D.); 5 Institute of Chemistry, Federal University of Uberlândia, Uberlândia-MG 38408-100, Brazil; 6 Federal University of Triângulo Mineiro, Iturama-MG 38025-180, Brazil

**Keywords:** electrochemical biosensor, visceral leishmaniasis, Chagas disease, gold nanoparticles, point-of-care, portable analysis

## Abstract

Visceral leishmaniasis is a reemerging neglected tropical disease with limitations for its diagnosis, including low concentration of antibodies in the serum of asymptomatic patients and cross-reactions. In this context, this work proposes an electrochemical immunosensor for the diagnosis of visceral leishmaniasis in a more sensitive way that is capable of avoiding cross-reaction with Chagas disease (CD). Crude *Leishmania infantum* antigens tested in the enzyme-linked immunosorbent assay (ELISA) were methodologically standardized to best engage to the sensor. The antibodies anti-*Trypanosoma cruzi* and anti-*Leishmania* sp. Present in serum from patients with diverse types of CD or leishmaniasis were chosen. A screen-printed carbon electrode modified with gold nanoparticles was the best platform to guarantee effective adsorption of all antigens so that the epitope of specific recognition for leishmaniasis occurred efficiently and without cross-reaction with the evaluated CD. The current peaks reduced linearly after the recognition, and still were able to notice the discrimination between different kinds of diseases (digestive, cardiac, undetermined Chagas/acute and visceral chronic leishmaniasis). Comparative analyses with ELISA were performed with the same groups, and a low specificity (44%) was verified due to cross-reactions (high number of false positives) on ELISA tests, while the proposed immunosensor presented high selectivity and specificity (100%) without any false positives or false negatives for the serum samples from isolated patients with different types of CD and visceral leishmaniasis. Furthermore, the biosensor was stable for 5 days and presented a detection limit of 200 ng mL^−1^.

## 1. Introduction

Leishmaniasis is a complex of diseases caused by a protozoan of the genus *Leishmania* [1,2] that affects millions of people worldwide. Visceral leishmaniasis, caused by *Leishmania donovani and Leishmania infantum*, represents the most severe form and can lead to death if not treated [3,4]. The onset of the infection and clinical manifestations are dependent on many factors including environmental and host immunologic status, especially in the early stages of infection [5]. Visceral leishmaniasis represents a major health problem in some tropical areas of the world. The currently available serum diagnosis does not fit the proper criteria of sensitivity and specificity, especially for identification of asymptomatic and or low symptomatic patients due to the low concentration of antibodies in the serum, particularly in the case of asymptomatic patients, which results in high cross-reactions [6]. Furthermore, due to its epidemiological characteristics, a diagnostic test that is accessible in remote areas is a desired tool for precise diagnosis and early therapeutic intervention.

The diagnosis of visceral leishmaniasis is made by combining clinical signs with parasitological or serological tests; however, they depend on extremely equipped laboratories, qualified labor, and a long period of time to carry out the tests [7,8]. The parasitological diagnosis is the reference choice for exams for detecting the disease, which shows the parasite directly in tissues or in culture. Aspirates from the spleen, bone marrow, and lymph nodes are used, and liver biopsy can also be performed. These techniques have high specificity and variable sensitivity. Until 2014, the Ministry of Health used two tests: the fluorescence indirect antibody test (IFAT) for human leishmaniasis, developed by the Institute of Immunobiological Technology (Biomanguinhos), Fundação Oswaldo Cruz, Brazil, and the Kalazar Detect rapid test (InBios International, Seattle, WA, USA).

Several studies show a comparison between the techniques, such as rapid test rK39, direct agglutination test (DAT), and ELISA, with some even showing good sensitivities and specificities in these patients [9]. However, the identification of asymptomatic infection remains challenging, since it depends on the sensitivity and specificity of the employed technique [10]. Furthermore, there is no agreement among the available techniques, and thus it is necessary to search for the best method to measure visceral leishmaniasis positives not only in symptomatic patients [11]. Hence, the average linear range of asymptomatic patients is a concern.

In this context, the presentation of new methodologies that show good performance, easy handling, speed, and detection of asymptomatic patients is essential to the control and early treatment of leishmaniasis. In other words, the development of tools that contribute to the optimization of a portable platform of leishmaniasis is a priority. Thus, the development of electrochemical biosensors shows advantages in comparison to traditional techniques, such as fast execution, a small amount of sample utilization, portability, selectivity, and specificity to obtain diagnoses.

The physical principle of the biosensor is to turn the biological sign into an electrical sign, making it possible in this way to monitor and quantification of signals. The immobilization of antigens, which specifically recognize the antibodies, can be provided on the surface of the sensor. The biorecognition, in the case of the evaluated pathologies, involves the antibodies as the target of detection, and they can be also quantified [9,12].

Electrochemical biosensors were developed by some research groups seeking to solve the problem of diagnosis of visceral leishmaniasis, investigating novel platforms and modification procedures for the diagnostic needs of *Leishmania* sp. [13,14,15,16,17,18,19,20,21,22,23]. Amongst the platforms used in sensors, the carbon-based electrodes present conditions for immobilization (through adsorption) once they enable a random anchorage and orientation of the biomolecules on its surface. Meanwhile, gold electrodes enable oriented couplings that minimize the distance between the active biomolecule sites and the electrode surface, facilitating the electron transfer, and a greater number of antibodies can be immobilized on the electrode surface [24,25,26]. Considering the use of portable platforms for point-of-care diagnosis, screen-printed electrodes play a key role in the development of electrochemical biosensors for several applications, as reported in the literature [27,28,29,30,31,32,33,34,35].

In order to an immunosensor functions properly for the diagnosis of leishmaniasis, it should be highly sensitive, specific, fast, and simple, with potential application for the serological diagnosis of leishmaniasis, since the disease is directly related to cross-reactivity with Chagas disease and other diseases. There are some studies reporting sensors for the diagnostic of *Leishmania* sp. Infection. Mohan et. al. [36] developed a genosensor modified with NiO nanostructured on an indium-tin oxide conductive glass plate to distinguish DNA from parasites extracted from human DNA extracts; however, these tests have not yet been performed in clinical samples. Moradi et al. [13] also developed a genosensor based on gold nanoparticles immobilized on polycrystalline gold discs and found high sensitivity in cutaneous leishmaniasis. However, the DNA extraction process has a higher cost compared to the use of protein antigens. Facing the diagnostic difficulties of cross-reactions, in this study, we describe how the electrode allows for differentiated interactions of the same biomolecule using a gold nanoparticle-modified surface, starting from the principle of this being biologically compatible and non-toxic. Such properties have attracted attention in diagnostic application because the molecules should not have their biological properties altered [26,36,37].

In this context, this work shows the development of an immunosensor for the specific diagnosis of visceral leishmaniasis without the cross-reactivity with Chagas disease (CD). Linear response, sensitivity, selectivity, specificity, repeatability, reproducibility, and stability were researched. To our knowledge, the concentration of *Leishmania* antigens considered as potential risk to develop the disease is not accurately known, and for this reason the proposed biosensor provides a detection limit in very concentrations (ng mL^−1^) to detect antigens before the appearance of disease symptoms.

## 2. Materials and Methods

### 2.1. Reagents and Biomolecules

All used reagents were of analytical grade and were used without further purification. Ultrapure water (MilliQ, Resistivity value greater than 18.2 MΩ, Millipore Corporation, Burlington, MA, USA) was used in the preparation of all solutions. The aqueous solution of the mixture of potassium ferricyanide/ferrocyanide ([Fe(CN)_6_]^3−^/[Fe(CN)_6_]^4−^) in KCl (5 mmol L^−1^, 0.1 mol L^−1^, pH 7.4, LabSynth, Brazil) used for the electrochemical characterization of the immunosensor was prepared immediately before the use. Gold (III) chloride was dissolved in sulfuric acid medium (1 g L^−1^ AuCl_3_ in 10 mL of 0.5 mol L^−1^ sulfuric acid). All experiments were carried out at controlled room temperature (25 ± 1 °C).

Preparation of the leishmania infantum crude antigen: The PP75 strain of *Leishmania infantum* cultured in the Schneider medium, supplemented with 20% fetal bovine serum, in the exponential phase was centrifuged 2000× *g* at 25 °C for 20 min and then washed three times with phosphate buffer (PB) solution and discarded supernatant. The pellet was resuspended in PB containing 0.05% NP40 (Nonidet P-40 Substitute, Roche) with the COMPLETE protease inhibitor (ROCHE, SWI). The antigen was obtained by the method of freezing in liquid nitrogen and thawing in a 37 °C water bath and then centrifuging it at 10,000× *g* for 30 min, and then the supernatant containing the soluble crude antigen was stored at −80 °C until the moment of use. The protein concentration of the antigen was determined by the Lowry method [38]. Aliquots of the extract were stored with the total soluble antigens at −80 °C until further use. The preparation of crude antigens specific to *Leishmania infantum* was performed. This species is characteristic of visceral leishmaniasis [39]. The technique was performed and adapted on the basis of [40]. Moreover, in our experiments, the efficiency of soluble and membrane-free extract of the parasite (data not shown) was proven, since these *Leishmania infantum* antigens are able to specifically be recognized by the serum antibodies of patients with visceral leishmaniasis antibodies.

The real samples used in the experiments appeared favorable and were substantiated from CEP (Comitê de Ética em Pesquisa/Research Ethics Committee) by Plataforma Brasil. *Leishmania* sera have the CAAE 58301516.8.0000.5154 and were 1,846,584 in number. Chagas sera have the CAAE 64048117.3.0000.5154 and were 2,163,043 in number. The stock solutions of total antigens (0.01 μg mL^−1^) and serum (visceral leishmaniasis = 1:100–0.202 mg mL^−1^; CD = 1:100–0.146 mg mL^−1^) were diluted in deionized water and frozen until the electrochemical experiment.

### 2.2. Devices

Screen-printed carbon electrodes (DPR-110) and screen-printed gold electrodes (DPR-220 BT) were purchased from DropSens (Oviedo, Asturias, Spain), which consist of a ceramic strip containing a three-electrode system (working, counter, and reference electrodes) for a single-drop analysis. The reference was made of a silver ink (known as silver pseudo-reference electrode) and the counter and working electrodes were made of carbon ink (in DPR-110) or of made of a gold ink (in DPR-220 BT). The working electrode of the screen-printed carbon electrode was modified with gold by electrodeposition (next described), which is the third electrochemical device evaluated in this work. Electrochemical analyses were performed by cyclic voltammetry using Em Stat 1 equipment (PalmSens BV, The Netherlands) connected to a notebook. The changes in the electrochemical signals of [Fe(CN)_6_]^4−^/[Fe(CN)_6_]^3−^ (5 mM) were evaluated (scan rate of 100 mV s^−1^). For ELISA tests (EnSpire/PerkinElmer), optical density (OD) values were determined on a microtiter plate reader at 490 nm.

### 2.3. Indirect ELISA

The indirect ELISA for the detection of immunoglobulin G (IgG) antibodies against leishmania used high affinity plaques (Thermo Scientific Tm Nunc Tm, Waltham, MA, USA), which were sensitized with the antigens (1 μg mL^−1^), diluted in 0.06 mol L^−1^ carbonate-bicarbonate buffer (pH 9.6), and incubated for 18 h at 4 °C. After this period, all plates were washed six times with PB containing 0.05% Tween 20 (PB-T) and blocked with PB containing 5% skimmed milk powder (Molico, Nestle, São Paulo, Brazil—PB-M5%) for 4 h at room temperature. After further washing, the serum samples were 1:40 diluted in 5% PB-M and incubated for 2 hours at room temperature. After six washes, the anti-human IgG antibody (1:2000) conjugated to peroxidase (IgG/horseradish peroxidase(HRP), Dako) was added and incubated for 2 h at room temperature. After further washing, the reaction was developed by addition of the enzymatic substrate 1,2-orthophenylenediamine (OPD, Dako) with 0.05% H_2_O_2_ and stopped with H_3_PO_4_. Positive and negative controls were included on the plate. The levels of antibodies were expressed in ELISA, according to the following formula: EI = Abs sample/cut-off, where cut-off is calculated as the mean of the Abs of negative control serum plus three standard deviations. EI values > 1.4 were considered positive.

### 2.4. Electrodeposition of Gold Nanoparticles on Carbon Electrodes

The electrodes were submitted to a 30-cycle cyclic voltammetry (CV) pre-treatment in 1 mol L^−1^ H_2_SO_4_ solution in the potential range between −0.3 and +1.2 V at 100 mV s^−1^ for surface cleaning and activation. After that, the electrodes were submitted to the deposition of gold nanoparticles by 15 voltammetric cycles in a gold chloride (HauCl_4_, 1 g L^−1^) solution prepared in 1 mol L^−1^ H_2_SO_4_ in the potential range between 0.3 and +1.0 V at scan rate of 0.1 V s^−1^ [41,42,43,44]. After the electrodeposition, it is possible to verify the color change of the working electrode evidencing the formation of gold nanoparticles.

The activation of the modified electrode was performed by 10 cycles in 1 mol L^−1^ H_2_SO_4_ solution to eliminate impurities that can hinder the adsorption of molecules, diminishing the reproducibility and stability of the modified surface [45,46,47].

### 2.5. Immunosensor

**Figure 1:**
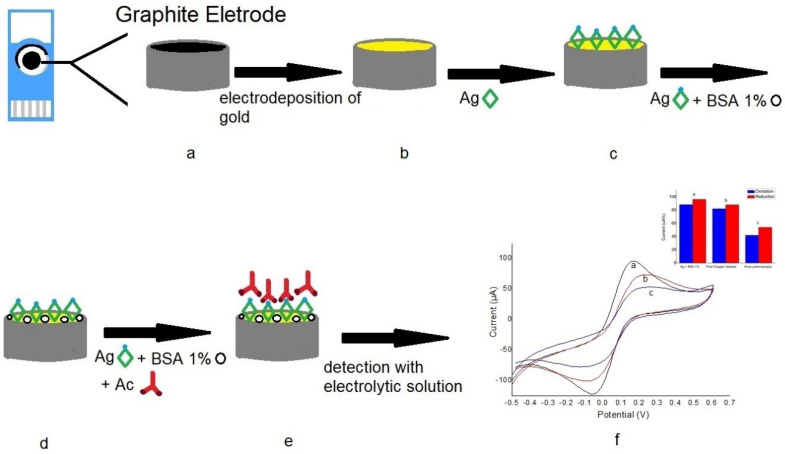
Scheme of the carbon immunosensor using a gold-modified electrode. The surface antigen probe was autonomized as presented in a voltammogram. The steps of preparation of the immunosensor are as follows: (**a**) the carbon electrode was selected as the base platform; (**b**) carbon was electrodeposited with gold nanoparticles; (**c**) the *Leishmania infantum* antigen was immobilized on the gold nanoparticle-modified surface; (**d**) the 1% bovine serum albumin (BSA) blocking solution was coupled to the platform as a blocking solution; (**e**) I after the preparations, the antibodies were coupled (real sample/serum); and (**f**) at the end, the electroanalytical solution was inserted and the process of transduction was initiated.

The first step involved the immobilization of the total soluble antigens on the surface of the working electrode (carbon, gold, and carbon modified with an electrodeposited gold nanoparticles) by drop-casting. The dissolution method of 4 μL lasted until the solution dried (15 min). In order to prevent nonspecific binding, we added 4 μL of 1% bovine serum albumin (BSA) as a blocking solution (15 min) after the first step. At the end, the serum was made available until it dried. After each step, the electrodes were washed and dried in a desiccator. For the interaction investigation between total antigens immobilized on each working electrode and total soluble antigens recognition, we used the solution of [Fe(CN)_6_]^4−/^[Fe(CN)_6_]^3−^ (5 mM) as a redox probe indicator. Thus, 80 μL of this solution was dropped over the three electrodes, closing the working electrode circuit between the other two electrodes (counter electrode and reference). Reactions occurred at room temperature (25 ± 1 °C). Using the cyclic voltammetry (CV) technique, we evaluated the behavior of the electrochemical signal of the supporting electrolyte (indirect detection) on the sensor, as shown in Figure 1.

### 2.6. Specificity

An aliquot of 4 μL of positive serum for Chagas disease (1:100 diluted) was pipetted on the immunosensor and kept for 15 min at room temperature. Thereafter, a final wash occurred with MiliQ water (50 μL), and the electrode was dried. Using the other electrode, the same protocol with positive serum for leishmaniasis (1:100 diluted) was performed, and on a third electrode, it was performed with serum negative (diluted 1:100, protein concentration in 5 μg mL^−1^). In all tests, including triplicates, the changes in the electrochemical signals of [Fe (CN)_6_]^4−^/[Fe (CN)_6_]^3−^ (5 mM) were evaluated (scan rate: 100 mV s^−1^).

### 2.7. Sensor Stability

To evaluate the stability of the immunosensor, we stored modified electrodes containing the total soluble antigen at 4 °C for 5 days, protecting them from light and oxygen.

### 2.8. Calibration Curve

To validate the immunosensor sensitivity analyses, we added 4 μL of different serum dilutions (1:25, 1:50, 1:100, 1:250, 1:100,000) to the immunosensor. Incubation was for 15 min at 25 ± 1 °C.

### 2.9. Statistical Analysis

The analyses are descriptive and are based on the comparative study of the voltammograms and their reinterpretations in bar charts and linear graphs (calibration).

## 3. Results and Discussion

### 3.1. Screen-Printed Electrode

**Figure 2:**
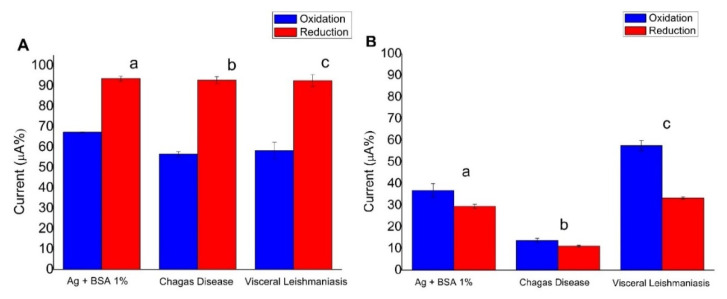
Column graphs extracted from cyclic voltammetry (CV) shows the variation of current peak percentages for (a) after immobilization of total soluble antigen followed by the addition of BSA, (b) after addition of Chagas disease (CD) antibodies, and (c) after addition of visceral leishmaniasis antibodies. The percentages were calculated from initial CV (without biomolecule) counting 100%. (**A**) Screen-printed carbon electrode used as a platform. (**B**) Screen-printed gold electrode used as a platform. The data are oxidation in blue and reduction in red. The electrochemical probe was 5 mmol L^−1^ [Fe (CN)_6_]^4−^/[Fe (CN) _6_]^3−^ and scan rate was 100 mV s^−1^.

Considering the differential molecular interaction with electrodes, we proposed the evaluation of the antigens and their recognition by anti-*Leishmania* antibodies present in the serum using a screen-printed carbon electrode and a screen-printed gold electrode (results shown in Figure 2A,B). It is important to emphasize that the same antibody/antigen system was used in the proposed electrochemical biosensor. The analyses enabled the evaluation of which platform increased stability and maintenance of the biological activity of the antibody because the immobilization of the probe on the electrode surface is a crucial step in the development of the sensors. To homogenize the analyses, we assembled the column graphs from current peak data, extracted from CV measurements. The current percentages (oxidation and reduction currents of the redox probe) were calculated from the initial CV (without the biomolecule) counting 100%. As the immobilization of biomolecules occurs by physical adsorption, conducting sites of the working electrode are blocked and therefore a fall of the current occurs (fewer conducting sites are available for the redox probe undergoes the electron transfer). Thus, low percentage refers to high blocking surface due to proportional immobilization or molecular recognition (Figure 2). On carbon (Figure 2A), an affinity for adsorption of antigens was shown, but no recognition for antibodies present in serum from patients with CD and visceral leishmaniasis was found, which indicates the effect of cross-reactivity. On the other hand, when the gold electrode was used as a platform (Figure 2B), a greater affinity for CD occurred more effectively in comparison with visceral leishmaniasis. This result may be explained by the molecular organization of the antigens on the gold surface due to thiol group presented in the leishmaniasis antigens, as previously stated in the literature for the immobilization of visceral leishmaniasis antigens [48].

### 3.2. Gold-Modified Electrode Used as a Platform

**Figure 3:**
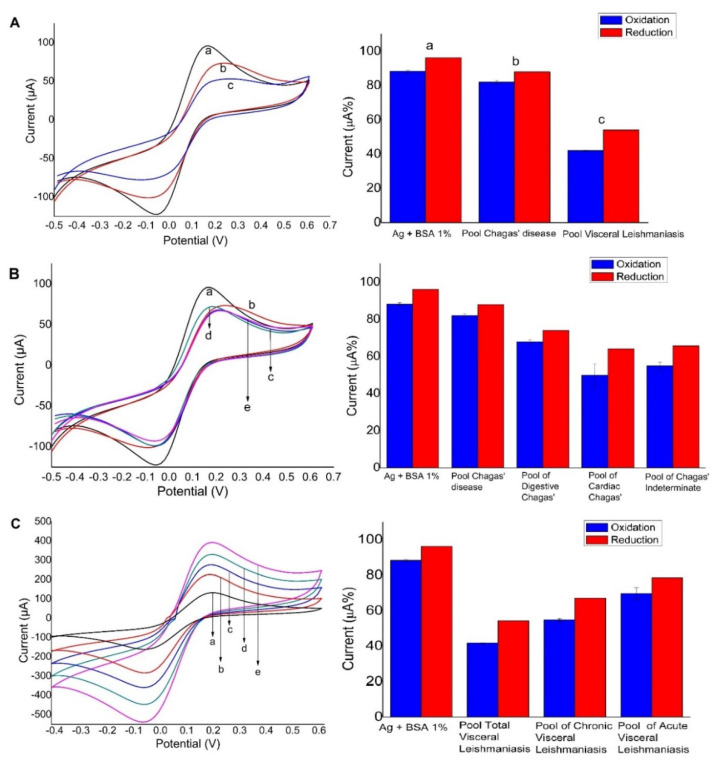
Cyclic voltammograms for the redox probe on the carbon electrode modified with gold nanoparticles that were electrodeposited and antigens; bar plots show the variation of peak current percentages. (**A**) Immunosensor (a), pool Chagas serum (b), pool visceral leishmaniarum (c). (**B**) Immunosensor (a) and different clinical forms by CD: Chagas serum pool (b), cardiac Chagas serum (c), digestive Chagas serum (d), Chagas undetermined serum (e). (**C**) Pool of total visceral leishmaniasis (a), pool of acute visceral leishmaniasis (b), pool of chronic visceral leishmaniasis (c), antigen *Leishmania infantum* + BSA1% (d), pool negative for leishmaniasis (e). The changes in the electrochemical signals of [Fe (CN)_6_]^4−/^[Fe (CN)_6_]^3−^ (5 mM) were evaluated (scan rate of 100 mV s^−1^).

Preliminary results using the screen-printed gold electrodes showed higher sensitivity than the screen-printed carbon electrodes; however, the results were not completely satisfactory when the immunosensor was evaluated in real samples (sensitivity was still moderate), and for this reason we investigated a novel platform—the gold-modified screen-printed carbon electrodes. This platform is well known for the formation of gold nanoparticles by electrodeposition [41], and thus the working electrode of the screen-printed carbon electrode strip was used as the electrode surface for modification. The modification with gold nanoparticles provided an increase in surface area and hence could potentially improve sensitivity. Figure 3A shows the CV recordings and the respective current percentages (bar plot beside the CVs) after the addition of (a) antigens, (b) CD, and (c) visceral leishmaniasis. This figure shows that the affinity for adsorption of antigens was effective and even more effective for the recognition of anti-leishmania antibodies than using the gold electrode because the current values of the redox probe decreased sequentially in (b) and (c). We can note the absence of cross-reactivity with Chagasic serum, proving the specificity of this sensor (Figure 3A). Moreover, on the basis of the higher efficiency of antigen immobilization without affecting its biorecognition site, we selected carbon electrodes modified with electrodeposited gold nanoparticles. Figure 3A also shows that the total soluble antigens could quantitatively discriminate (b) and (c) from each serum (CH 146, LSH 202). Figure 3B shows the response in the presence of groups of different clinical forms of Chagas diseases. Even under these conditions, the immunosensor responded only to anti-*Leishmania* antibodies, which indicated the absence of cross-reactivity. Figure 3C shows changes in current peaks from the different types of leishmaniasis, indicating the recognition of the immunosensor towards the different leishmaniasis antibodies.

### 3.3. Stability

The stability studies of the biosensor were evaluated under storage at 4 °C for 5 days, protected from light and oxygen. This experiment was performed with the same electrode previously optimized using a gold-modified screen-printed carbon electrode modified with leishmaniasis antigens. The biosensor was evaluated in the presence of anti-*Leishmania* antibodies and we observed a decrease of 46% in the detection capacity after 5 days. This signal decrease indicated lack of stability, which is a compromising feature of the proposed biosensor. Future experiments are required to investigate a condition to improve the stability of the immunosensor.

### 3.4. Calibration Curve

**Figure 4:**
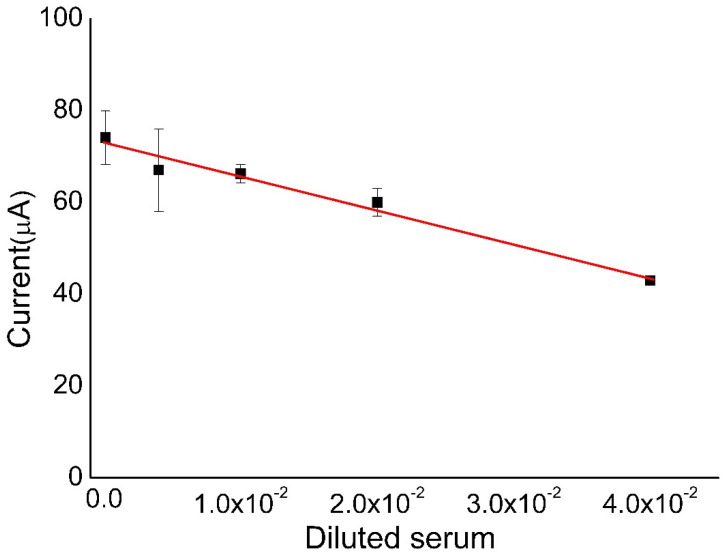
Calibration curve obtained from current peak percentages for triplicate measurements of the biosensors in the presence of diluted serum (1:25, 1:50, 1:100, 1:250, and 1:100,000) containing antibodies (stock serum solution of 20.2 mg mL^−1^). The percentages were calculated from initial CV (without biomolecule) counting 100%. The changes in the electrochemical signals of [Fe(CN)_6_]^4−^/[Fe(CN) _6_]^3−^ (5 mM) were evaluated (scan rate of 100 mV s^−1^).

Figure 4 shows the preliminary analysis of the calibration curve using the immunosensor. Keeping in mind that the current of the redox probe is inversely proportional to the concentration of antibodies, we used diluted serum at 1:25, 1:50, 1:100, 1:250, and 1:100,000 ratios. Higher dilutions did not generate linearity on the results (tests performed in triplicates). This plot presents the correlation coefficient of 0.9746 (for the equation: i(%) = −727.5 × [serum dilution ratio] + 72.83), an estimated limit of detection of 202 ng mL^−1^, and limit of quantification of 606 ng mL^−1^. The inset shows the equation obtained from the linear regression of a current peak (%) vs. concentration of leishmaniasis.

In the screen-printed electrodes without alteration of their surfaces (carbon surface), it was possible to observe that there was reactivity and absence of specification. The choice of electrodeposition of gold nanoparticles provided to the carbon electrode new physical-chemical properties of the biomolecules that were immobilized on its surface. The results demonstrated that the electrodeposition of the gold nanoparticles was not only capable of promoting reactivity, but also the desired selectivity. In addition to improving the responses on biosensor platforms, it also allowed the interaction of the biological probes to the surface of the same ones [49]. Given the results, the electrodeposited gold on the screen-printed carbon electrode improved the sensitivity of the sensor by effectively increasing the surface area of the electrode, promoting a greater site of adsorption of total soluble antigens [50]. Moreover, the orientation of the total soluble antigen adsorption on gold nanoparticles may have contributed to the improved specificity not obtained when the total soluble antigens were immobilized on the unmodified carbon electrode. The interaction and orientation of total soluble antigens may occur through the S–H bonds of some amino acids of the total soluble antigens, knowing that these were strongly linked to gold (chemisorption/covalent) [51,52]. This combination generates a late rally organized system between the biomolecules in a spontaneous, stabilized, and oriented way, being well-known as a self-assembled monolayer [48]. This kind of modification has extra experimental advantages over the use of gold in biosensors. Furthermore, it is linked to ease of handling and preparation, low cost, accessibility, and stability without the need for an additional step involving the addition of a thiol monolayer on the electrode surface [53,54].

### 3.5. Comparison with Indirect ELISA

**Figure 5:**
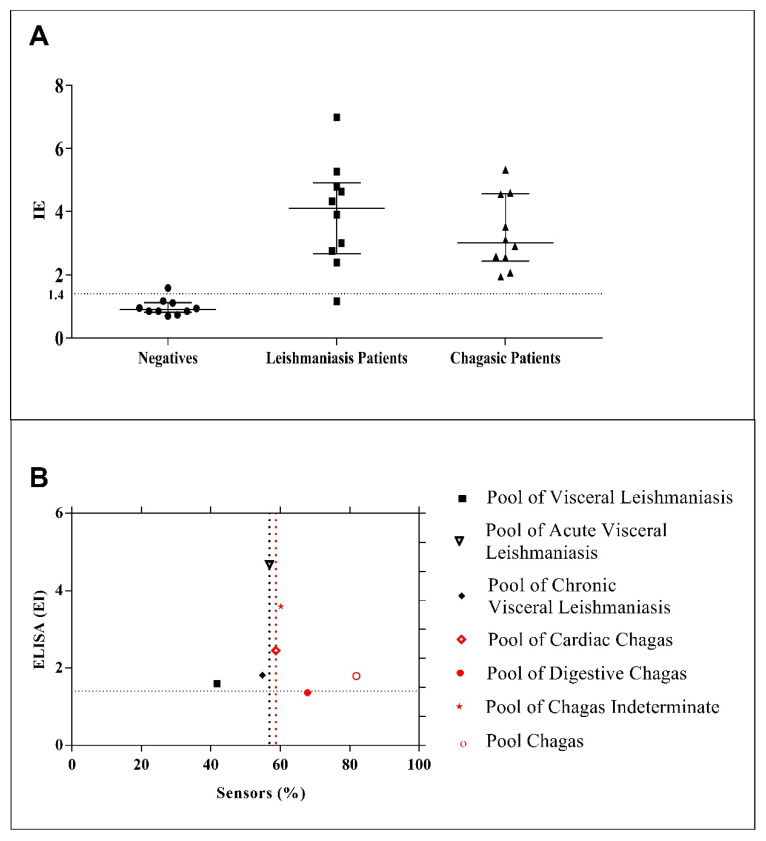
(**A**) Distribution of ELISA index (EI) values obtained by indirect ELISA (data from Table S1), with a line at EI = 1.4 to guide the reader (values above 1.4 are considered positive for the tests), for serum samples from patients with different types of diseases. (**B**) EI values (ELISA) and percentage current (proposed immunosensor) obtained for the same serum samples of different diseases (acute and chronic visceral leishmaniasis; cardiac, digestive, and indeterminate Chagas; and a pool of all). Current values below the black dotted line indicate positive tests for leishmaniasis, while current values above the red dotted line indicate negative tests.

In this study, a comparison of the results of the electrochemical immunosensor with indirect ELISA was carried out. The different diagnostic methods of visceral leishmaniasis present detection difficulties justified by the occurrence of cross-reactions with other trypanosomatids, explained by phylogenetic limitations existing among protozoa [1]. The results for indirect ELISA indicate low specificity (Table S1 shows the representation values from the ELISA plate tests shown in Figure S1). As expected, the cross-reaction occurred, with discrimination only in positive serum from patients with acute leishmaniasis (Figure S1). The lack of specificity that occurred in the ELISA test can be explained by the occurrence of affinities between the total antigen of visceral leishmaniasis and the antibody of Chagas disease. Figure 5A shows the distribution of ELISA index (EI) values obtained for the tests performed in different serum samples (data from Table S1), with the individual tests showing cross-reaction. From the distribution in Figure 5A, it is possible to observe 1 false negative and 15 false positive tests. Figure 5B shows the EI values and percentage current obtained by the proposed immunosensor obtained for the same serum samples (six different diseases and a pool of all of them). We found that the proposed biosensor detects Chagas disease as being a weak interaction, with this connection being due to the existence of some possible interactions between the total leishmaniasis antigen and the anti-*Trypanosoma cruzi* antibody that causes Chagas disease. In the detection of leishmaniasis, there is a strong interaction between the total visceral leishmaniasis antigen and the anti-*Leishmania infantum* antibody, a result that converges with what is expected in theory. While the electrochemical immunosensor was able to discriminate acute and chronic leishmaniasis from all the analyzed serum samples, the ELISA test showed values of EI higher than 1.4 for almost all cases presented in Figure 5B, which indicates false negative for cardiac, digestive, and indeterminate Chagas. Thus, the electrochemical biosensor can differentiate diseases, even with the occurrence of such affinities reported in the literature.

On the basis of these results, we calculated the selectivity and specificity parameters for the electrochemical biosensor and ELISA using the following equations [55,56]:

(Specificity) = (total of negative tests)/(false positives + total negative tests)(1)

(Sensitivity) = (total of positive tests)/(false negatives + total positive tests)(2)

ELISA tests presented low specificity (44%) for a total of 38 serum samples analyzed due to the high number of false positives (15). Selectivity was calculated as 93.2% due to one false negative test.

On the other hand, the proposed electrochemical biosensor did not present any false positive or false negative for the total of 12 analysis of serum samples from patients of different types of CD and leishmaniasis, and consequently the calculated specificity and sensitivity values were both 100%. Therefore, the developed immunosensor presented impressive advantages over the current ELISA tests for the diagnosis of *Leishmania infantum*.

Finally, Table 1 compares the sensitivity and specificity of the proposed electrochemical biosensor and ELISA tests with data referring to the diagnostic tests of existing leishmaniasis. This table shows that the proposed biosensor presents the highest sensitivity and specificity values compared to traditional ELISA tests, as well as other diagnostic tests.

## 4. Conclusions

The results showed that the electrodeposition of gold nanoparticles on the carbon electrode was efficient in immobilization of the antigens, providing better results than bare carbon electrode or gold electrode. This property allowed specific epitope recognition of antibodies present in serum from patients with visceral leishmaniasis. It should be noted that the use of serum from patients with CD solves a major problem in detection tests present in the market. We aim to improve and develop these sensors for application in public health, that is, in a large number of tests with high performance in sensitivity and specificity. Therefore, future studies will be extended in the selection of antigens purified for the diagnosis of asymptomatic visceral leishmaniasis patients and tests with extended periods in stability. In this way, such a tool may be feasible for practical applications and commercial purposes, with a reduced cost compared with Western blot, and improved performance in comparison with ELISA. Hence, electrochemical immunosensors offer great promises for specific and selective diagnosis of visceral leishmaniasis.

## Figures and Tables

**Table 1:** Results of research conducted on patients with the aim of finding an efficient diagnosis of leishmaniasis.

Method	Material	Sensitivity/Specificity	Reference
IFAT	Serum	88–92%/83–88%	[57]
Kalazar Detect	Serum	84–88.1%/91%	[57]
IT LEISHBio-Rad	Blood/Serum	92–93%/92–98%	[57]
PCR	Blood	93%/96%	[8,57,58]
DAT-LPC	Blood	99%/98%	[57]
RIFI	Serum	0–100%/80%	[9,10]
Immunochromatographic tests	Serum	87%/94%	[11,12,13]
ELISA	Serum	80–99%/81–100%	[14,15,16,17,18]
Electrochemical	DNA extracted of blood	Not explained	[22]
ELISA	Serum	92.3%/44%	This work
Electrochemical	Serum	100%/100%	This work

## Supplementary Materials

The following are available online at https://www.mdpi.com/2079-6374/10/8/81/s1, Figure S1: ELISA plates for immunological tests. Table S1: Values of ELISA index (IE) obtained for all samples. Values above 1.4 are considered positive for the tests.
