# Supplementary material for: Development of an Electrochemical Immunosensor for Specific Detection of Visceral Leishmaniasis Using Gold-Modified Screen-Printed Carbon Electrodes
Source: Biosensors (Basel). 2020 Jul 23;10(8):81. doi: 10.3390/bios10080081 (PMC7460044; doi:10.3390/bios10080081)
Supplement: Supplementary file 1 [file biosensors-10-00081-s001.pdf]

biosensors

Supplementary Material

Development of an Electrochemical Immunosensor for Specific Detection of Leishmaniasis using Gold-Modified Screen-Printed Carbon Electrodes

Beatriz R. Martins [1], Yanne O. Barbosa [1], Cristhianne M. R. Andrade [2], Loren Q. Pereira [2], Guilherme F. Simão [3], Carlo J. de Oliveira [1,2], Dalmo Correia [2], Robson T. S. Oliveira Jr [1], Marcos V. da Silva [2], Anielle C. A. Silva [4], Noelio O. Dantas [4], Virmondes Rodrigues Jr [1,2], Rodrigo A. A. Muñoz [5,*] and Renata P. Alves-Balvedi [1,6,*]

1 Institute of Biological and Natural Sciences, Federal University of Triângulo Mineiro, Uberaba-MG 38025-180, Brazil (UFTM); biaroma_95@hotmail.com (B.R.M.); yanne.way@hotmail.com (Y.O.B.); carlo.oliveira@uftm.edu.br (C.J.de O.); robson.junior@uftm.edu.br (R.T.S.O.Jr); virmondes.rodrigues@uftm.edu.br (V.R.Jr)
2 Institute of Health Sciences, Federal University of Triângulo Mineiro, Uberaba-MG 38025-180, Brazil (UFTM); cristhianne_m@hotmail.com (C.M.R.A.); lorenbiomedica@gmail.com (L.Q.P.); dalmo@mednet.com.br (D.C.); marcosuftm@gmail.com (M.V.da S.)
3 Institute of Technological and Exact Sciences, Federal University of Triângulo Mineiro, Uberaba-MG 38025-180, Brazil (UFTM)
4 Institute of Physics, Federal University of Alagoas, Maceio-AL 57072-970, Brazil (UFAL); acalmeida@fis.ufal.br (A.C.A.S.); noelio@fis.ufal.br (N.O.D.)
5 Institute of Chemistry, Federal University of Uberlândia, Uberlândia-MG 38408-100, Brazil (UFU)
6 Federal University of Triângulo Mineiro, Iturama-MG 38025-180, Brazil (UFTM)
* Correspondence: munoz@ufu.br (R.A.A.M.); renata.balvedi@uftm.edu.br (R.P.A.-B.)

ELISA Tests

Figure S1 shows ELISA plates for serum samples included isolated patients for acute Leishmaniasis (1, 7-10), Chronic Leishmaniasis (2-6), Chagas Digestiva (14), Chagas Cardiac (15-17, 19), Undetermined Chagas (11-13, 18, 20) and Negatives for Chagas and Leishmania (20-30). In addition, Pools were tested from patients of the above classifications, pools were obtained after a mixture of these diseases. Pool Leishmaniose Acute (1'), Pool Leishmaniose Chronic (2'), Pool Chagas Undetermined (3'), Pool Chagas Cardiac (4’), Pool Chagas Digestiva (5’), Negative Pool for Chagas and Leishmania (6'), Initial Pool Chagas (7'), Initial Pool Leishmaniose (8’), and blanks (B).

biosensors

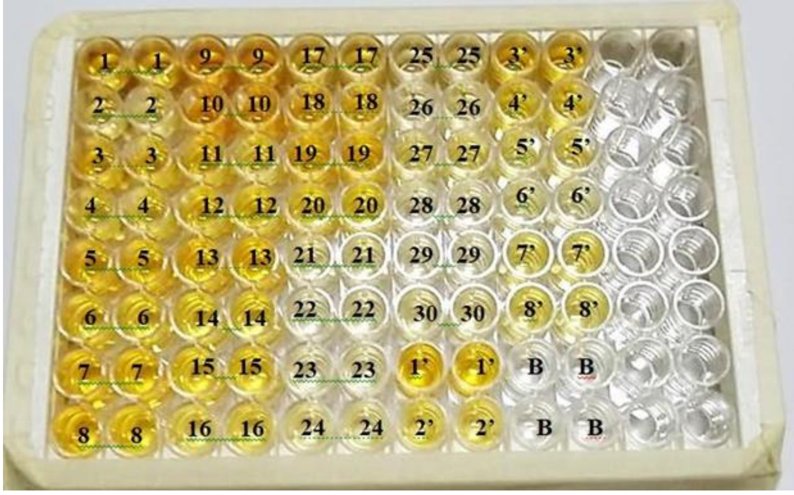

Figure 1. ELISA plates for immunological tests.

Table 1. Values of ELISA index (IE) obtained for all samples. Values above 1.4 are considered positive for the tests.

**A: IE**	**A: IE**	**A: IE**	**A: IE**	**A: IE**	**A: IE**	**A: IE**
1: 2.76	7: 4.63	13: 4.60	19: 5.33	25: 0.94	1′: 4.68	7′: 1.79
2: 1.16	8: 3.90	14: 2.58	20: 2.55	26: 0.85	2′: 1.81	8′: 1.59
3: 4.32	9: 5.27	15: 3.11	21: 0.95	27: 1.58	3′: 3.59	
4: 2.39	10: 6.98	16: 1.94	22: 0.85	28: 0.70	4′: 2.45	
5: 4.79	11: 2.91	17: 2.07	23: 0.85	29: 0.73	5′: 1.36	
6: 3.01	12: 3.52	18: 4.55	24: 1.17	30: 1.11	6′: 0.92	
